# The Application of Porphyrins and Their Analogues for Inactivation of Viruses

**DOI:** 10.3390/molecules25194368

**Published:** 2020-09-23

**Authors:** Natalya Sh. Lebedeva, Yury A. Gubarev, Mikhail O. Koifman, Oskar I. Koifman

**Affiliations:** 1 Laboratory 1-7. Physical Chemistry of Solutions of Macrocyclic Compounds, G.A. Krestov Institute of Solution Chemistry of the Russian Academy of Sciences, 153045 Ivanovo, Russia; gua@isc-ras.ru; 2 Department of Chemistry and Technology of Macromolecular Compounds, Ivanovo State University of Chemistry and Technology, 153000 Ivanovo, Russia; koifman@mail.ru (M.O.K.); koifman@isuct.ru (O.I.K.); 3 Laboratory 2-2. New Materials on the Basis of Macrocyclic Compounds, G.A. Krestov Institute of Solution Chemistry of the Russian Academy of Sciences, 153045 Ivanovo, Russia

**Keywords:** viruses, SARS-CoV, chlorin, DNA, porphyrins, phthalocyanines

## Abstract

The problem of treating viral infections is extremely relevant due to both the emergence of new viral diseases and to the low effectiveness of existing approaches to the treatment of known viral infections. This review focuses on the application of porphyrin, chlorin, and phthalocyanine series for combating viral infections by chemical and photochemical inactivation methods. The purpose of this review paper is to summarize the main approaches developed to date in the chemical and photodynamic inactivation of human and animal viruses using porphyrins and their analogues and to analyze and discuss the information on viral targets and antiviral activity of porphyrins, chlorins, of their conjugates with organic/inorganic compounds obtained in the last 10–15 years in order to identify the most promising areas.

## 1. Introduction

Over the past decades, the onset of epidemics and pandemics caused by SARS viruses (SARS-CoV, 2002–2003), swine flu (H1N1, 2009–2010), bird flu (H7N9, 2013), the Middle East virus (MERS-CoV 2013–2015), the Ebola virus (2014–2015), and the SARS-CoV-2 coronavirus conditioned the need to consider a healthcare system not only as a public sector organizing and protecting the health of the population of a particular country but also as a global international task. Change of priorities for threat evaluation takes place throughout a civilized society. The reason for this is the emergence of new viral infections that cause epidemics and pandemics, as well as the existence of drug-resistant infections, which are increasing each year [1,2,3,4]. Therefore, the search and development of new alternative approaches to the treatment of viral and bacterial infections are necessary. The proposed review will consider the main achievements over the past few years in the search for alternative therapeutic approaches and of the drugs based on porphyrins and their analogues (P&A) for the treatment of viral infections.

For an objective and comparative analysis of existing approaches and for revealing the most promising areas, we briefly consider the structure of viral particles. This will allow us to establish potential vulnerabilities, the so-called molecular targets.

Viruses are microscopic pathogens, translated from Latin word virus, which means “poison and poisonous source”. Mammalian viruses have various shapes, most often spherical or ellipsoidal with sizes ranging from 18 to 900 nm [5]. Viral particles contain genetic material in the form of DNA or RNA, often a protein shell (capsid), and may also include additional lipid shells (supercapsid or peplos). Viruses are obligate intracellular parasites. They are carriers of their own genome during intercellular distribution and transmission from organism to organism. They protect the genome during transit and, together with nonstructural proteins, deliver it to the cytosol or nucleus of the host cells; after that, the mechanism of replication and formation of daughter virions is triggered. As a rule, the penetration of the pathogen into the host cell leads to its death.

Viruses are diverse, and their various classifications exist. The International Committee on Virus Taxonomy (ICTV) [6] has concluded that virus taxonomy should include basic evolutionary relationships between remotely related viruses. The new release, dated March 2020, contains taxonomic changes approved by ICTV members. This issue includes in the system 4 realms, 9 kingdoms, 16 types, 2 subtypes, 36 classes, 55 orders, 8 suborders, 168 families, 103 subfamilies, 1421 genera, 68 subgenuses, and 6590 types of viruses. Despite their comparative simplicity, viruses are extremely diverse in their genetic material and replication mechanisms [7].

As a rule, the life cycle of a virus is a complex process, which is divided into the following stages: (1) attachment of the virion to the cell surface (linking of the host cell receptor); (2) internalization of the virus into the cell (endocytosis); (3) decapsidation, cytoplasmic transport, and nuclear imports; (4) transcription and replication of viral RNA/DNA; (5) nuclear export and protein synthesis; and (6) assembly and release of propagated virions from the host cell membrane.

All these stages of the life cycle of a virus are of great importance for its virulence, replication, and transmission. Violation of any of these can lead to the development of a potential and effective strategy for controlling and preventing viral infection. The inactivation of the first three stages of the life cycle of the virus has the greatest practical application, so this review will be focused on them.

## 2. Inactivation at the Stage of Attachment of the Virion to the Cell Surface (Receptor Binding) and Fusion with the Cell Membrane

**Figure 1:**
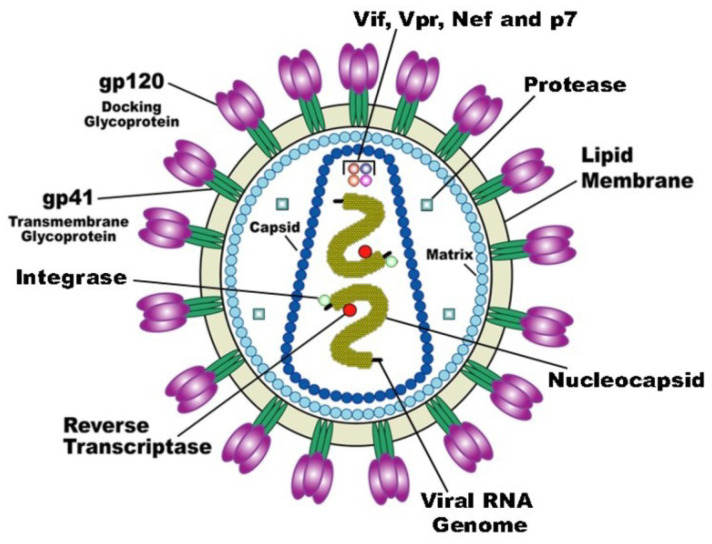
The structure of the HIV virion [8].

As noted above, despite the comparative simplicity, all 6590 types of viruses differ in their genetic material, replication mechanism, and in chemical structure of structural and nonstructural proteins. Therefore, an understanding of the biomechanics and biochemistry of the functioning of structural proteins of viral particles is necessary for a scientifically based search for virucidal compounds. Now, we will consider the methods of inactivating viral particles, using porphyrins and their analogues at the first stage of the life cycle, taking as the example human immunodeficiency virus (HIV). The vast majority of studies focus on HIV inactivation, partly because this virus appeared relatively long ago (in 1983) and has been well studied, but to a greater extent, owing to the slowly progressive disease caused by it, which often leads to death. According to WHO, there are about 40 million people diagnosed with HIV in the world. The human immunodeficiency virus belongs to the genus *lentiviruses* (Lentivirus) of the family of retroviruses (Retroviridae). HIV virus is relatively simple (Figure 1). In addition to structural proteins, it contains two RNA helices and three enzymes: reverse transcriptase (revertase), integrase, and protease.

**Figure 2:**
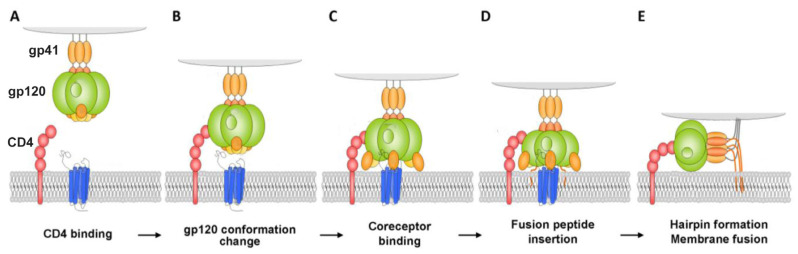
Model for HIV-1 entry. (**A**,**B**) Binding of cluster of differentiation (CD)4 to glycoprotein (gp)120 exposes a coreceptor binding site in gp120; (**C**,**D**) coreceptor binding causes the exposure of the gp41 fusion peptide and its insertion into the membrane of the target cell in a triple-stranded coiled-coil; and (**E**) formation of a helical hairpin structure in which gp41 folds back on itself is coincident with membrane fusion [9].

The first stage of attachment of the virion to the surface of the cell and fusion with the cell membrane is determined by the spikes on the surface of the virion. The spikes on the viral particle are formed by Env glycoprotein (envelope protein), which consists of two subunits: the variable protein gp1209 performing the function of binding to the CD4 receptor and of the more conservative transmembrane gp41, which holds the gp120 molecule in the membrane (Figure 1). The gp120 viral glycoprotein strongly links the CD4 receptor (Figure 2).

**Figure 3:**
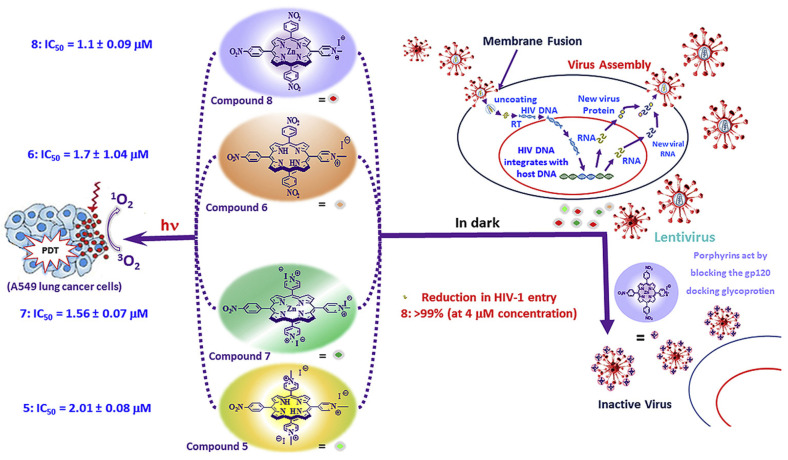
Scheme of the lentivirus life cycle and method of its inactivation by nitroderivatives of porphyrins [12].

As a result of this interaction, gp120 undergoes conformational changes that also provide binding the coreceptor molecule CXCR4 or CCR5 (Figure 2). This is followed by the stage of internalization of the virus into the host cell; the latter ensures the fusion of the cell membranes of the virus and the host due to the gp41 viral transmembrane protein. Thus, the proteins of the gp120 or gp41 virus are the most important and critical targets, since if they are damaged or linked, further propagation of the HIV virus becomes impossible. Among the porphyrins and their analogues, several very promising compounds have been identified that are capable of forming strong complexes with the gp120 protein glycoprotein [10], and the complex formed in this way does not allow the viral gp120 of protein to bind to the host cell CD4 receptor (Figure 3) [11].

## 3. Porphyrins and Their Analogues Structure

The structure of the porphyrins and their analogues plays a key role in linking gp120 protein with the glycoprotein as illustrated by the example of the compounds shown in Figure 3. It was found that the porphyrin–zinc complex containing three 4-nitrophenyl groups and one 4-methylpyridinium group in the mesoposition (Figure 3, compound **8**) most effectively blocks the penetration of virions (4 μM, >99%). A cationic substituent is necessary to increase the solubility of porphyrins and their analogues in aqueous media, and three nitro groups provide linking of the gp120 protein with the glycoprotein (Figure 3) and thereby inhibit fusion between the virus and the cell membrane.

**Figure 4:**
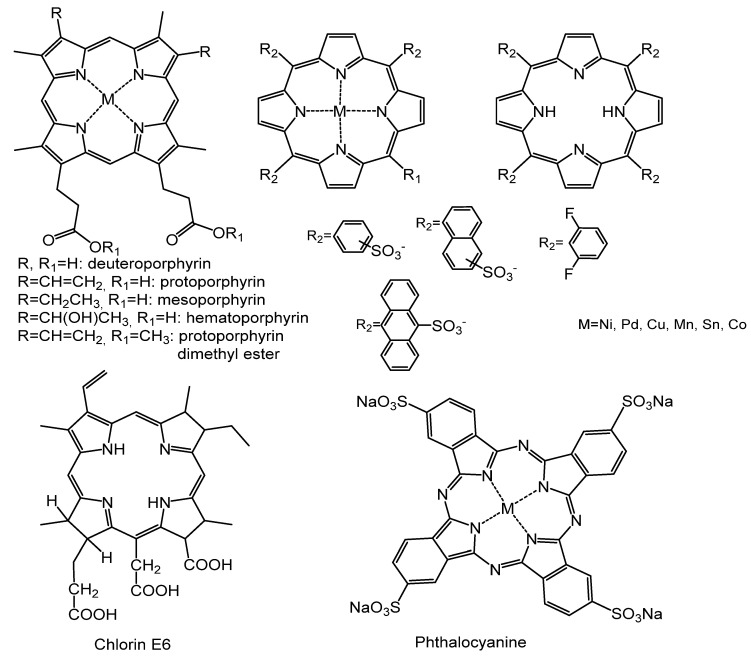
Structures of the compounds.

A wider range of porphyrins and their analogues, including deutero-, proto-, hemato-, and *meso*-porphyrins, derivatives of metal-tetraphenylporphyrin-tetrasulfonate and derivatives of sulfonated tetraarylporphyrins was tested for anti-HIV activity (Figure 4) [13].

Screening showed that none of the deutero-, proto-, hemato-, and *meso*-porphyrins reduced infection by more than 80% at a concentration of 5 μg/mL. The most active compounds were sulfonated tetranaphthyl-porphyrin, sulfonated tetraanthracenyl-porphyrin and sulfonated 2,6-difluoro-mesotetraphenylporphine and its copper complex. Their antiviral activity was 99%, 96%, 94%, and 96%, respectively. Such a high virucidal activity, according to the authors of [13], is due to the linking of HIV-1 gp120 with porphyrins and their analogues, and this reaction completely inhibits the ability of Env proteins to induce cell fusion with the receptors of CD4 host cells.

A comparison of the results of studies [12,13] allows us to conclude that the charge of porphyrins and their analogues is not determining in linking with viral gp120, since both cationic [12] and anionic [13] porphyrins showed a similar high virucidal activity. It can be assumed that the electrostatic interactions in the MGC-gp120 system are not significant, and it is logical, since the outer part of gp120 consists of sites that are very variable and carry multiple sugars (the basis for the virus to evade the immune response to this protein in the body). Detailed studies of the structure of the gp120 target showed that this protein contains five variable regions (V1–V5) with five conserved regions (C1–C5). The region involved in coreceptor linking has been identified as a polycation, which includes the V3 loop of the gp120 protein and conservative elements adjacent to this loop [14]. To target the V3 loop, porphyrins and their analogues must be negatively charged.

It should be noted that targeted modelling of the porphyrins and their analogues structure for linking with a specific region of the target has not yet been carried out. At this stage of development of this research direction, the porphyrins and their analogues are mainly screened, both using computer modelling [15] and experimental methods [16]. For example, the antiviral activity of methyl and metal-free porphyrin and chlorin compounds against a wide range of viruses with and without envelope was evaluated in [17]. Chlorophyllide was most effective against hepatitis B virus (HBV). Only 3 μM chlorophyllide reduced HBV DNA signal by 80%. To establish the mechanism of the virucidal activity of chlorophyllide, the authors conducted in vitro biochemical studies in which chlorophyllide was introduced on each life cycle of the virus. It turned out that chlorophyllide changed the structure of the capsid of HBV virions and directly destroyed the HBV virions in the culture medium of producer cells, and it resulted in the loss of DNA virion. Chlorophyllide does not have a noticeable effect on the viability of the host cells and intracellular products of viral genes; it is safe for humans at a dose of 300 mg/d for 4 months [18].

Chlorin e6 has demonstrated the strongest antiviral activity against hepatitis B virus as well as a deep antiviral effect on other enveloped viruses, such as hepatitis C virus (HCV), human immunodeficiency virus (HIV), dengue virus (DENV), Marburg virus (MARV), Tacaribe virus (TCRV), and Junin viruses (JUNV). It is noteworthy that chlorin e6 inactivated DENV at the subnanomolar level. However, the compound did not exert an antiviral effect against the enveloped virus of encephalomyocarditis virus (the genome of the virus is single-stranded plus-RNA) and adenovirus (DNA containing) [18].

Chemical inhibition at the stage of attachment of the virion to the cell surface is possible not only by the action of the porphyrins and their analogues on the viral protein but also by an alternative linking of the host cell receptor itself. For example, oxovanadium mesoporphyrin IX forms very stable complexes with the CD4 receptor, which in its stability compete with the best peptidomimetics (cyclometallic complexes of platinum (II)) [19].

All the studies reviewed above are based on the method of chemical inactivation of viruses. Without any doubt, this approach is promising, but in the formation of complexes between the target and the porphyrins and their analogues due to π–π-interaction, axial coordination, H-bonding, and electrostatic interaction, there always remains the option of destruction (collapse) of the complexes. In addition, it should be borne in mind that for a positive antiviral effect, the required amount of P&A can be significant. First, this follows from the basics of thermodynamics and is associated with the need for an excess of P&A to increase the number of the combined surface protein structures. Second, the number of spines themselves on the virion of viruses is very different e.g., on the surface of the HIV-1 virion there are about 7–14 spines (Env) [20], influenza virus has 400–500 spines, vesicular stomatitis virus has about 1200, and the more the spines present, the greater the number of porphyrins and their analogues required. Third, the problem of target linking selectivity arises, i.e., part of the porphyrins and their analogues can interact with other biosubstrates. In addition, the determination of the required dose of porphyrins and their analogues should be correlated with the degree of damage to the body. Finally, another equally important factor—like resistance to bacterial antibiotics—is the resistance to antiviral drugs that also remains an urgent issue [21], because with chemical inhibition, the virus still exists, it is only inactivated. In this sense, the use of photoinactivation of viral particles seems more promising, since photoinactivation involves the complete destruction of the viral infection.

**Figure 5:**
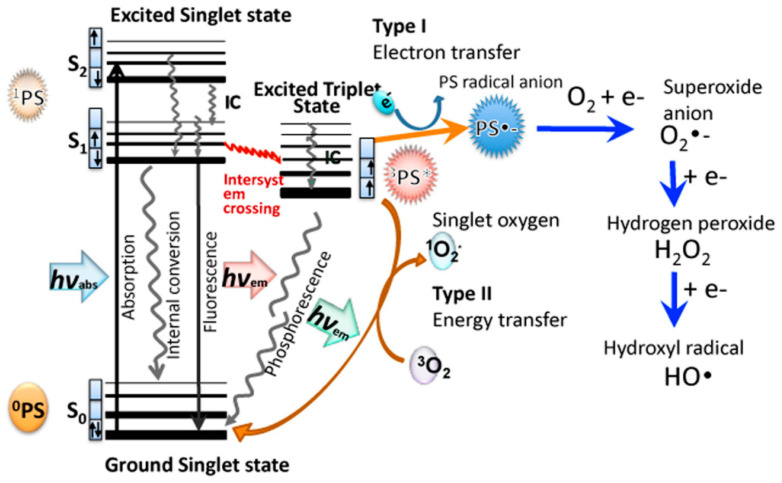
Jablonski diagram [23].

It is well known that porphyrin, chlorin, and phthalocyanine series is capable of absorbing light energy and generating reactive oxygen species. This process can proceed according to two mechanisms—type I and type II [22], the essence of which is as follows: in the ground state, the porphyrins and their analogues are a singlet, the absorption of a photon of light leads to the excitation of one electron and its transition to an orbit with a higher energy (Figure 5).

The singlet state is short-lived (nanosecond lifetime), and excess energy can be emitted by the P&A in the form of light (fluorescence) or dissipated into the energy of disordered processes and, ultimately, emitted into heat. The third option, “intersystem crossing,” is also possible, in which the P&A goes into a more stable excited triplet state (microsecond lifetime). From the point of view of photochemistry, triplet state allows the P&A to transfer energy upon contact with molecular oxygen (O_2_); as a result, the P&A returns to its original singlet state, and oxygen passes into the excited triplet state (^1^O_2_). The described photochemical reaction is called the type II photochemical process [24]. A type I photochemical process can occur when a P&A photosensitizer in an excited state undergoes electron transfer reactions, which ultimately lead to the formation of reactive oxygen species (ROS). Their formation occurs as follows: at the first stage, electron transfer is carried out with the formation of a cation/anion radical. A radical anion can react with oxygen to form a superoxide anion radical (O_2_^•−^). The dismutation or one-electron reduction of O_2_^•−^ produces hydrogen peroxide (H_2_O_2_), which, in turn, can undergo another one-electron reduction with the formation of hydroxyl radicals (HO^•^) [22].

Active oxygen species obtained during photoexposure to the P&A will react with the immediate environment of the P&A and lead to irreversible chemical changes in the target.

According to a number of researchers [22,25], the majority of P&As used in photoinduced oxidative processes of biomolecules “operate” via a type II mechanism. This opinion is based on a comparison of the rate constants of energy transfer processes in the production of ^1^O_2_ (k ≈ 1–3 × 10^9^ dm^3^·mol^−1^·s^−1^) and electron transfer in the production of O_2_^•−^ (k ≤ 1 × 10^7^ dm^3^·mol^−1^·s^−1^) [26]. According to other researchers, both mechanisms proceed simultaneously, and the contribution to the photooxidation of each of them depends on the nature of the P&A, the excitation wavelength [27], the duration and intensity of photoirradiation [28], and on other external conditions.

As noted above, all ROS have a short lifetime and high reactivity that results in damage of the immediate environment of the P&A. For the unenveloped viruses, capsid proteins, including host cell recognition proteins, are direct targets. For the enveloped viruses, the main targets are enveloped glycoproteins, including host recognition proteins, as well as the lipid membrane [29]. Moreover, in both cases, structural proteins play a decisive role in the interaction of the host virus and in the internalization of the virus into the host cell. Therefore, their irreversible photochemical damage can lead to the destruction of the virus.

The principal possibility of virus inactivation in photoinduced capsid damage was demonstrated on the bacteriophage MS2. The bacteriophage MS2 is often used as a model of the virus due to its nonpathogenicity to humans and ease of handling [29,30,31,32]. It turned out that 5,10,15,20-tetrakis-(*N*-methyl-4-pyridyl)porphin (TMPyP4) with a concentration of 0.2 μM destroys the bacteriophage under 1-min illumination with a power of 32 mW·sm^−2^. The authors attribute such rapid inactivation [29] to the oxidation of one of the sites of the viral protein responsible for the attachment of MS2 to the bacterial pilus and the delivery of its protein genome to the host genome. This site is enriched with amino acids residues of histidine and tryptophan known as amino acids of the increased vulnerability to ^1^O_2_ (by the rate of the chemical reaction between ^1^O_2_ and amino acids, the most vulnerable acids cysteine, methionine, tryptophan, tyrosine, and histidine are revealed (k = 8.9 × 10^6^, 1.6 × 10^7^, 3.0 × 10^7^, 8 × 10^6^ and 3.2 × 10^7^ М^−1^·с^−1^, respectively) [33]. The opposite conclusion was reached in [31,34], where it was shown that the complete cleavage of the capsid of the bacteriophage MS2 occurred due to the oxidation of Cys101, which is the only susceptible and accessible ^1^O_2_ amino acid residue in this peptide.

One cannot fail to note the research [35] in which the photoinduced TMPyP4 inactivation of four x-shell-free RNA viruses (bacteriophages MS2 and Qβ, bovine enterovirus (BEV-2), and mouse norovirus (MNV-1)) were studied. Viruses virions are of the similar size in 27–35 nm and contain icosahedral capsids. This work is noteworthy because the authors tried to establish a relationship between the rate of photoinactivation of the virus and the content of vulnerable amino acids in the capsid protein (histidine, tryptophan, methionine, and cysteine), and they determined the possibility of the effect of photooxidation on the inner surface of the capsid protein and on the viral genome. For icosahedral viruses, the phenomenon of capsid respiration is known [36], so the question if photoactivation can affect the viral genome has some sense. It turned out that RNA extracted from virion susceptible to photooxidation and transfected into RAW 264.7 cells exhibited titers compared to viral RNA not subjected to photooxidation. The obtained results confirm that in this case, two targets were hit during TMPyP4 photoactivation: capsid protein and RNA.

Cationic tetrakis(*N*-methyl-4-pyridiniumyl) porphyrin, tetrakis(*N*-[*n*-butyl]-4-pyridiniumyl) porphyrin, tetrakis(*N*-[*n*-octyl]-4-pyridiniumyl) porphyrin, and anionic porphyrins (tetrakis(4-sulfonatophenyl) porphyrin) were evaluated as potential drugs for inactivation of nonenveloped viruses (hepatitis A virus and bacteriophage MS2). The authors set themselves the task of determining the effect of the charge and hydrophobicity of the P&A on its virucidal activity [37]. Unfortunately, the quantum yield of singlet oxygen and SOR was not taken into account in this work; therefore, the conclusions drawn need to be revised. A similar error was made in [28], aimed at establishing the effect of the P&A charge on the photoinactivation of a T4-like bacteriophage.

Enveloped viruses such as vesicular stomatitis virus (VSV) are photoinactivated by hydrophobic P&As (blood type porphyrins). The envelope of the vesicular stomatitis virus is a lipid bilayer obtained from the host cell; it contains trimers of one type of integral glycoprotein, called the G-protein, which allows the virus to enter the target cell through endocytosis, and it catalyzes the fusion of viral and endosomal membranes. Dose-dependent inactivation of the virus was enhanced during photoactivation of porphyrins (protoporphyrin IX, Zn (II) protoporphin, mesoporphyrin IX) [38]. It turned out that all three porphyrins intercalate into lipid vesicles and disrupt the structure of the viral membrane. In addition, these photoporphyrins caused viral glycoproteins VSV to crosslink. Incubation of viruses with sodium azide and α-tocopherol partially protected VSV from inactivation by porphyrins, and it indicates that the process proceeded according to type II.

Heme, cobalt protoporphyrin IX (CoPPIX), and tin protoporphyrin IX (SnPPIX) display antiviral activity against arboviruses, such as Dengue virus, yellow fever virus, Zika virus, Mayaro virus, Sindbis virus, and dental vesicular virus. Porphyrin treatment results in the loss of viral enveloped protein, affects the morphology of the virus, adsorption, and also blocks penetration into host cells. Photoirradiation enhanced SnPPIX activity against all tested arboviruses [39].

Another example of photoinactivation of a virus with an envelope by porphyrin, the HIV virus, is presented in [40]. The authors, taking into account the positively charged V3 loop of the gp120 protein, selected a series of anionic porphyrins containing two sulfonate and two carboxyl groups with different degrees of esterification as photosensitizers. In the dark phase, the esterified porphyrin compounds inhibited anti-V3 antibodies, but combined with the conserved region of C5. After photoirradiation, inhibition of antibodies of the C5 region was exclusively observed. The results are rather unexpected, as it is reliably known that ROS are very reactive and short-lived [41]. According to the evaluations of [42], the lifetime of ^1^O_2_ in water is 4 μs, while its maximum possible path does not exceed 150–220 nm in the absence of quenchers [43]. In the system under consideration, judging by the data of [40], porphyrins are localized in region V3, and they cause photodamage in region C5. The authors explained this unusual phenomenon by the presence of a large number of vulnerable amino acids in the C5 protein fragment.

Speaking about the photodynamic inactivation of viruses with the envelope, one cannot fail to note the work [44] devoted to the creation of influenza drugs. Influenza virus (type A, B, and C) is one of the main human pathogens responsible for respiratory diseases, causing high morbidity and mortality from seasonal flu. The influenza virus is a (−)RNA virus. A virion consists of a core, a shell, and matrix proteins. These proteins are hemagglutinin (HA), neuraminidase (NA), matrix protein 1 (M1), proton channel protein (M2), nucleoprotein (NP), RNA polymerase (PA, PB1, and PB2), nonstructural protein 1 (NS1), and nuclear export protein (NEP, NS2). The RNA genome of the influenza virus constantly mutates with the formation of new viral subtypes. Although a vaccine is the most effective way to prevent influenza, vaccine formulations should be updated annually due to changes in circulating influenza viruses, and it takes several months to produce a flu vaccine. If the prognosis of incoming influenza strains is wrong, vaccines can offer only limited protective effectiveness [45].

**Figure 6:**
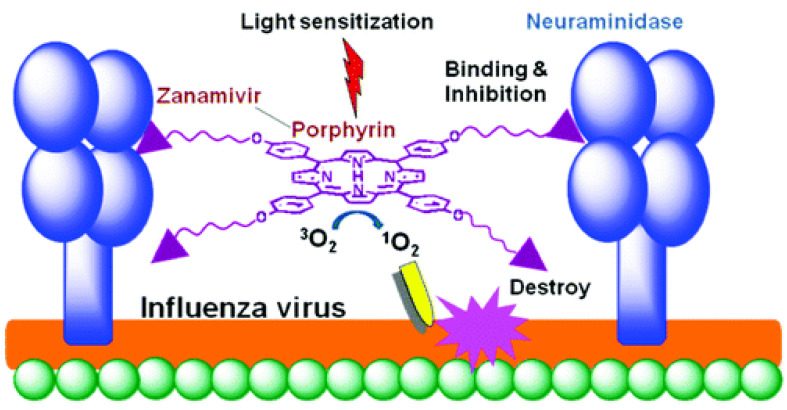
Schematic representation of the life cycle of an influenza virus [46,47].

The stage of attachment of the virion to the cell surface (receptor binding) and fusion with the cell membrane is mediated by hemagglutinin (HA), which interacts with the host cell glycoproteins containing terminal sialic acid (*N*-acetylneuraminic acid, Neu5Ac) [46,47] (Figure 6). Thus, hemagglutinin is a target for inactivating the influenza virus.

**Figure 7:**
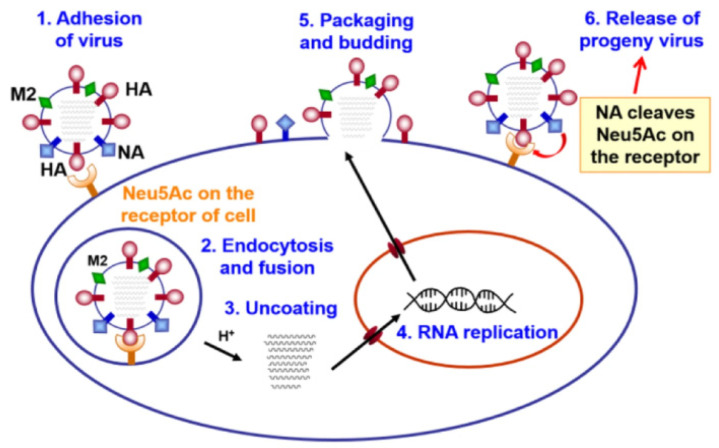
“Magic bullet” approach for inactivation of influenza viruses [44].

A synthetic approach was developed to create a conjugate of porphyrin with zanamivir. The optimal length of the spacer was determined to ensure retaining of the affinity of zanamivir (Figure 7) [44]. Zanamivir due to its high affinity for the hemagglutinin of the virus promoted targeted delivery of porphyrin, and porphyrin generated photocontact with singlet oxygen, which oxidized viral neuraminidase. The photochemical oxidation of this enzyme did not allow it to catalyze the hydrolytic reaction of cleavage of the terminal residue of Neu5Ac from the sialo-receptor on the surface of the host cell (for the release of new virions). It should be noted that this is one of the few works in which the construction of the P&A photosensitizer for inactivation of the virus was carried out.

Some studies have reported that photoactivation of viruses without a membrane is faster and with a lower amount of P&A photosensitizer [48] than viruses with a membrane. Other works make the opposite conclusions [49], e.g., cationic tetraplatinized porphyrins do not exhibit any virucidal activity against enveloped viruses, but they effectively inactivate enveloped viruses (BVDV and BoHV-1), which cause cattle diseases.

**Figure 8:**
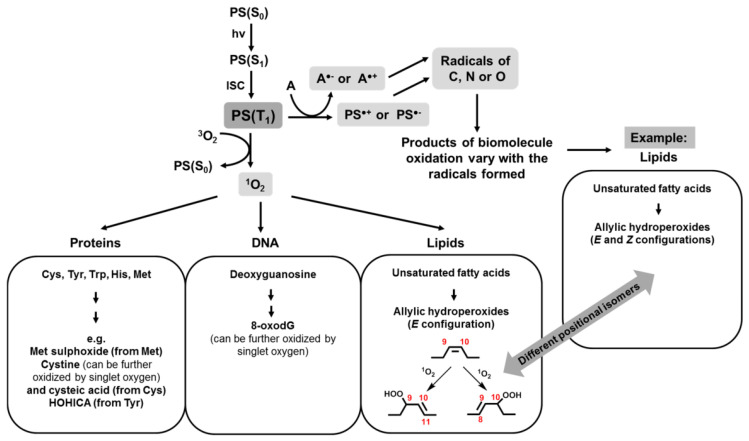
Main routes and initial products of singlet oxygen- and radical-mediated photooxidations [25].

It is probably not correct to compare the virucidal activity of P&As with respect to enveloped and unenveloped viruses, since: (1) a priori, it should be different P&As, in the first case, they should be hydrophobic for localization in lipid structures, whereas in the second one, more hydrophilic; (2) different P&As will have different ROS quantum yield; (3) capsid and supercapsid possess different local oxygen concentration; and (4) photoinduced damages in the capsid and supercapsid are different [25] (Figure 8).

These damages are revealed to a greater extent on protein than in lipid structures [25]. Protein oxidation can lead to a change in protein conformation, oxidation of amino acid residues [26,50], crosslinking, and destruction of the main chain of the polypeptide up to the capsid cleavage. There are several approaches to studying the state of viral proteins after photoinitiated reactions. These approaches are the traditional polyacrylamide gel electrophoresis (SDS-PAGE), IR spectroscopy [51], mass spectrometry with matrix laser desorption ionization (MALDI) [31], and ionization by sputtering in an electric field (ESI) [26,31], and the method based on the use of antibodies [29], as well as the classical biochemical method for evaluating the virucidal effect of the oxidation process by means of cultivation procedure. The use of SDS-PAGE and IR spectroscopy provides general information about the state of viral proteins, their aggregation and cleavage, but it is impossible to obtain a detailed information on the affected domains, on oxidation of side chains, loops, and on conformation changes in the secondary and tertiary structure of the protein. The advent of mass spectrometric methods provided accurate mass measurements of whole viral particles and individual capsid proteins before and after oxidation. These methods make it possible to determine the degree of fragmentation of the main chain of the polypeptide and to identify areas of damage of the protein capsid, of photo-induced dimerization, and aggregation, i.e., those processes that lead to significant changes in the molecular weight of the protein. Protein oxidation can also result in structural changes in the side chains of amino acid residues, to transformations that have only a moderate effect on the mass of the protein but can have dramatic consequences for its functioning. Antibodies can help in detecting even small damaged areas [29].

Lipid oxidation causes less dramatic changes if the photoinduced process is carried out according to the type II mechanism. Then singlet oxygen reacts with unsaturated lipids with the formation of lipid hydroperoxides (LOOH) and it leads to a change in the thickness, area [52], and permeability of the lipid layer [53,54]. In the case of oxidation by mechanism I, i.e., when free radical mechanisms are involved in the first stage, the hydrogen atom is cleaved from unsaturated fatty acid (LH) and carbon-centered lipid radical (L^•^) is formed, which, when interacting with an oxygen molecule, gives the radical LOO^•^. The LOO^•^ radical is capable of reacting with other LH fatty acids and this reaction leads to the formation of lipid hydroperoxide (LOOH) and another lipid radical (L^•^) [54].

Thus, numerous studies have shown that P&A compounds can act as virucidal chemotherapeutic and photoinactivating compounds at the stage of attachment of the virion to the cell surface (receptor binding) and fusion with the cell membrane, regardless of the presence or absence of supercapsid. It is likely that the degree of virulence of the P&A will depend significantly on the localization of the P&A and the selectivity of linking with a target. For targeted modelling of the MGC structure, reliable information on the structure of the target is needed. There is a definite correlation between virucidal activity and chemotherapeutic activity and photoinactivating ability. As a rule, P&As exhibiting both of the latter properties are virucidal [55,56].

## 4. Inactivation at the Stage of Transcription and Replication of Viral RNA/DNA

As noted above, HIV virion contains three enzymes: reverse transcriptase (RNA-dependent DNA polymerase), which is involved in the synthesis of DNA on an RNA matrix; an integrase that catalyzes the incorporation of the generated viral DNA into the chromosome of the host cell; and a protease that splits synthesized polyproteins into structural proteins. The stage of transcription and replication of viral RNA/DNA is impossible without the participation of these enzymes which are the target for chemotherapeutic agents used in the treatment of AIDS.

In this context, a publication [57] should be noted in which it was reported that a new site for the reverse reductase enzyme has been identified, and it was capable of binding compounds of the porphyrin class. To identify it, the authors compared the protein sequences of reverse reductase and heme-binding proteins and detected an identical conserved site (398–407 position in the HIV-1 reverse reductase polypeptide chain (WETWWTEYWQ) [58]. This site contained an excessive amount of aromatic amino acid residues which, according to the authors of [57], led to high stability of the complexes of this site with iron(III) and zinc(II) protoporphyrins (the binding constant is about (1.3 ÷ 4) × 10^5^ dm^3^·mol^−1^). It should be noted that the binding of protoporphyrins to the indicated site by metal complexes was not highly selective, and the antiviral activity exhibited was relatively low (10 μm, >80%).

The significance of the nature of the metal complexing agent in the processes of HIV inactivation is evidenced by the results of [59]. According to them, reverse transcriptase is inhibited by porphyrins containing aminosulfonyl peripheral substituents. In this case, the metal-free porphyrin and its zinc complex were active at 33% and 6.4% inhibition, respectively, while the vanadium complex of porphyrin showed much better results (5 μM with an inhibition of more than 97%). However, as shown in [57], porphyrins demonstrated a low selectivity for reverse transferase binding. The selective binding of reverse transferase with iron-protoporphyrin dimethyl ether, protoporphyrin dimethyl ether, and its sodium salt has been established [60], but the virucidal activity of natural porphyrins has not been evaluated.

Unfortunately, it was not possible to find publications in which highly selective binding of P&A to reverse transferase was achieved due to the introduction of peripheral substituents containing an oligopeptide sequence complementary to the HIV fragment, although this approach is used to increase the tumorigenicity and selectivity of the P&A used in PDT. For example, porphyrin conjugates can be synthesized, the peripheral substituents of which contain the HIV peptide sequence [61], and the latter provides targeting of cancer cells.

**Figure 9:**
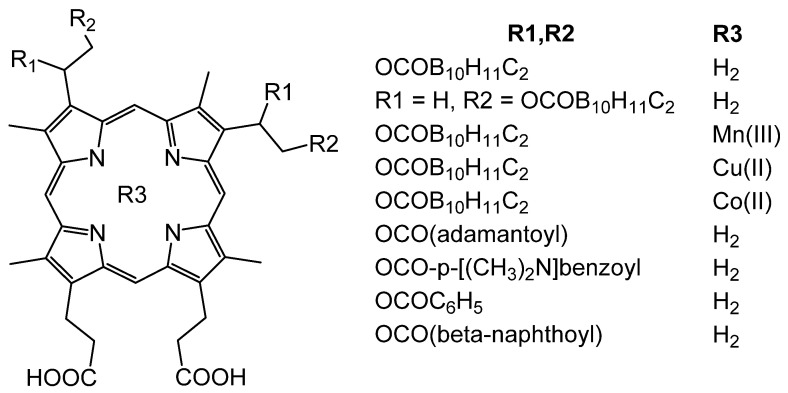
Scheme of porphyrin derivatives of HIV protease inhibitors.

Analysis of nonpeptide compounds with useful pharmacological properties synthesized for neutron capture therapy revealed several porphyrins containing carborane esters at positions 2 and 4 (Figure 9); these can inhibit micromolar amounts of HIV-1 and HIV-2 protease [62].

Porphyrin compounds containing carborane esters as peripheral substituents have a greater inhibitory effect on the protease compared with protoporphyrin IX and porphyrins not containing carboranes. The introduction of metals, both coordinatively saturated and unsaturated, negatively affects the ability of the P&A to inhibit the protease. Important factors are not only the presence of carborane groups but also their isomerism. The introduction of a methyl group into carborane also significantly reduces the affinity of P&A linking with the protease. Apparently, carborane substituents specifically interact with HIV protease, and it leads to a high affinity between the P&A and the enzyme. Replacing carborane cells with similar in size, but less hydrophobic groups, such as benzoyl, adamantoyl, β-napthoyl, allows one to obtain inhibitors in the low micromolar range, and it indicates the importance of hydrophobic interactions in stabilizing the complex of the P&A with the enzyme. The best result was obtained with porphyrin-tetrakiscarborane carboxylate ester of 2,4-bis-(α,β-dihydroxyethyl) deuteroporphyrin IX which is a submicromolar HIV protease inhibitor [62]. Moreover, for HIV-1 protease, the IC50 value is 185 nm, whereas for HIV-2, it is 700 nm.

The stage of transcription and replication of viral RNA/DNA is naturally impossible without the source of genetic material. Viral genomes contain DNA or RNA, which can be either double-stranded or single-stranded, linear, or circular in topology, and monocistronic or polycistronic. Genomes can be divided into several segments [5]. Reflecting the diversity of genetic material and replication mechanisms, Baltimore’s classification divides viruses into seven groups: double-stranded (ds) DNA genomes (group I), single-stranded (ss)DNA genomes (group II), dsRNA genomes, (group III), ss(+)RNA genomes (group IV), ss(−)RNA genomes in (group V), ssRNA genomes with reverse transcription of the dsDNA replication of intermediate (group VI), and dsDNA genomes with the ssRNA replication intermediate (Group VII) [63]. The genome of the vast majority of viruses has been decoded and can be found in specialized literature. Obviously, this genetic material of the virus is a supertarget for the fight against viral infection. Moreover, the linking of the P&A with the genetic material of the virus and its further photoinactivation are very promising.

**Figure 10:**
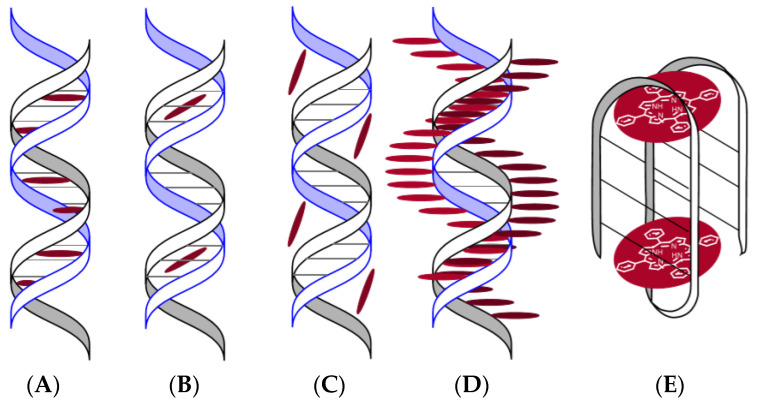
Schemes of complexes of P&A with DNA (**A**–**D**) and with G-quadruplexes (**E**) [64]. Internal complex (**A**), binding to a small groove of DNA (**B**), binding to a large groove of DNA (**C**), external binding with self-stacking along the DNA surface (**D**), and binding to a G-quadruplex (**E**).

Porphyrins and their analogues can form various types of complexes with DNA: intercalates (internal complex) (Figure 10A) [64], binding to a small groove of DNA (Figure 10B) [64], binding to a large groove of DNA (Figure 10C) [64], and external binding with self-stacking along the DNA surface (external complex) (Figure 10D) [64].

The way of linking porphyrins with DNA depends on the nature of the peripheral substituents of the P&A, on the metal of the complexing agent, the type of DNA [65,66,67], external conditions, and even the concentration of the P&A [64]. One of the first cationic porphyrins studied in terms of interaction with DNA was TMPyP4. The first publication dated 1973 [68] reported on the spectral manifestation of the interaction of TMPyP4 with DNA. Subsequently, the data were supplemented, and it was found that intercalation of porphyrin in DNA leads to a bathochromic shift of the Soret band (>20 nm) in the UV–Vis spectrum of porphyrin, to a decrease in its intensity by (~40%), while in the spectra of circular dichroism of the intercalate in the Soret region, the negative induced signal is recorded. The formation of an external complex (Figure 10D) is spectrally discovered in the bathochromic shift of the Soret band to 9 nm and a slight decrease in intensity (~5–7%) [64] is observed. The spectrum of circular dichroism of the complex (Figure 10D) includes one positive band in the Soret region [64]. In a number of works, it is noted that a positive induced CD band in the Soret region is indicative of external (minor) groove binding. In the case of the formation of an intercalate (Figure 10A), the interaction between porphyrin and a pair of nitrogenous bases guanine-cytosine is most likely [69,70]. During the formation of the complex (Figure 10D), the π–π interaction is realized between the aromatic system of the porphyrin macrocycle and the adenine–thymine pair. The spectral changes listed above are called “fingerprints”, i.e., they allow one to identify what type of complex is being formed (Figure 10).

When changing the composition of the medium or the molar ratio R (R ratio is the ratio of the concentration of DNA base to porphyrin, [DNA]/[P] ranges), the internal complex can transform into the external [64,67,71]. As a rule, the formation of intercalation complexes requires the planar structure of the P&A, small peripheral substituents, and the P&A:DNA ratio (for nitrogen base pairs) of less than 1:4. Cationic P&As are preferred since phosphate groups of DNA are negatively charged. The formation of intercalation of metal complexes of the P&A with DNA containing unsaturated or multiply charged metals is difficult or does not occur at all, due to steric hindrances caused by the existing axial ligand or metal counterion [64].

The thermodynamic stability of the complexes of P&A with DNA depends not only on the nature of P&A but also on the type of complex formed [71]. This condition is important when using P&A as a chemotherapeutic agent, as well, as it is shown below, when P&A is applied as a photoinactivator.

**Figure 11:**
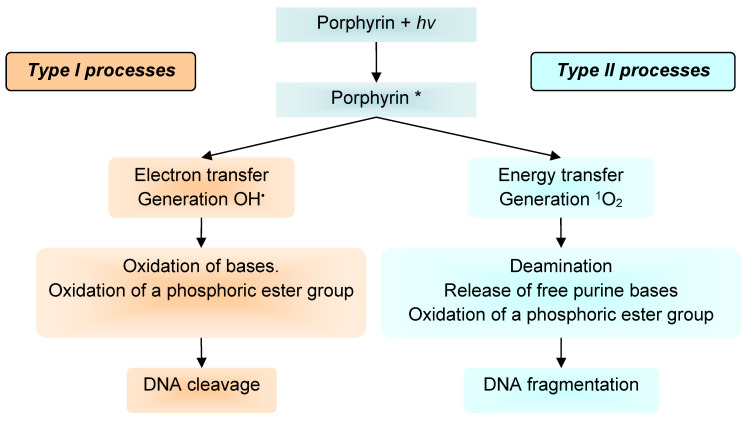
Scheme of DNA photoreactions of type I and type II. * excided triplet state.

As our own [64] and literary studies [72,73,74] have shown, depending on the type of photochemical process I or II (Figure 11), different results of DNA photooxidation can be obtained. The process according to mechanism I leads to oxidation of bases, oxidation of a phosphoric ester group, and, as a consequence, DNA cleavage [75]. The process according to mechanism II causes deamination, release of free purine bases, and oxidation of a phosphoric ester group and leads to DNA fragmentation [64,75,76].

**Figure 12:**
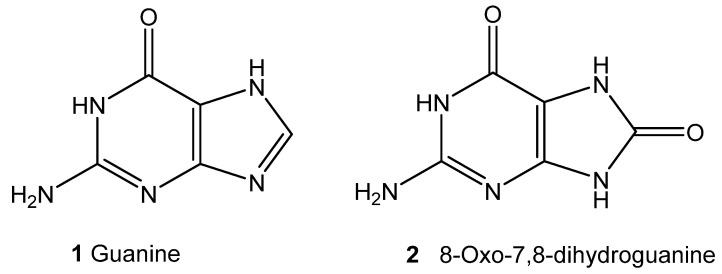
Guanine **1** and its oxidation product **2** [77].

In particular, the guanine fragments (Figure 12, compound **1**) are susceptible to oxidative damage, resulting in the formation of 8-oxo-7,8-dihydroguanine (Figure 12, compound **2**), one of the main products of type I photosensitized oxidation.

The result of photoirradiation of complexes of P&A with DNA substantially depends on the type of complex being formed. For example, in [64], it was shown that irradiation of porphyrin intercalates (TMPyP3 and TMPyP4) with DNA leads to DNA fragmentation, and in the case of TMPyP4, DNA fragments of different sizes are formed. Irradiation of external complexes of porphyrins with DNA results in the cleavage of DNA 5,10,15,20-tetrakis-(*N*-methyl-3-pyridyl)porphin (TMPyP3) and TMPyP4 and is accompanied by photolysis of porphyrin (TMPyP3).

According to the researchers [78], the binding of porphyrins TMPyP4 and *meso*-tri-(4-*N*-methylpyridyl) monophenylporphyrin (TMPyMPP) to DNA is not a prerequisite for photoinactivation. The authors of [78] consider that free porphyrins are more effective in inactivating the virus than DNA-related species and explain it by a lower quantum yield of singlet oxygen of the P&A in the complex as compared with free P&A [32]. This conclusion contradicts the generally accepted view that the P&A should be in close proximity to the place of photodamage. The reason for the controversial conclusions may be that the authors did not take into account the very probable [78] inversion of the intercalation complex into the external complex [64]. Having obtained intercalates at the initial stage, then increasing the concentration of porphyrins, they mistakenly believed that saturation of the intercalation complex was achieved and unbound porphyrin was present in the solution [78]. It is possible to achieve the formation of only one type of complexes with DNA due to a significant increase in the volume of peripheral porphyrin substituents, making intercalation sterically impossible. This was demonstrated in [79] by the example of oligo- and polypeptide conjugates of cationic porphyrins, which form exclusively external complexes with DNA.

In addition to the type of complex formed, an important influence is exerted by the intensity and duration of irradiation—these conditions are described in detail in the review [32].

Prevention of viral replication and reduction of virulence after DNA damage by active oxygen forms, generated by the P&A, was shown in [16,32,75,80]. As noted above, the genetic material of the virus can be represented by DNA and RNA. The most serious human diseases caused by RNA viruses are Ebola hemorrhagic fever, SARS, COVID-19, rabies, influenza, hepatitis C and E, West Nile fever, poliomyelitis, measles, etc. The viral genome can be represented as single-stranded (ssRNA single-stranded) and double-stranded RNA (dsRNA double-stranded) [81]. Single-stranded RNAs in accordance with their polarity are divided into single-stranded RNA viruses with a negative strand ((−)RNA) and single-stranded RNA viruses with a positive strand ((+)RNA). Single-stranded (+)RNA acts as a messenger RNA and can be directly translated by an infected cell (it can individually cause an infectious disease). Therefore (+)RNA is also called semantic. Antisemantic (−)RNA of the virus before translation must be converted to (+)RNA by the action of RNA polymerase.

**Figure 13:**
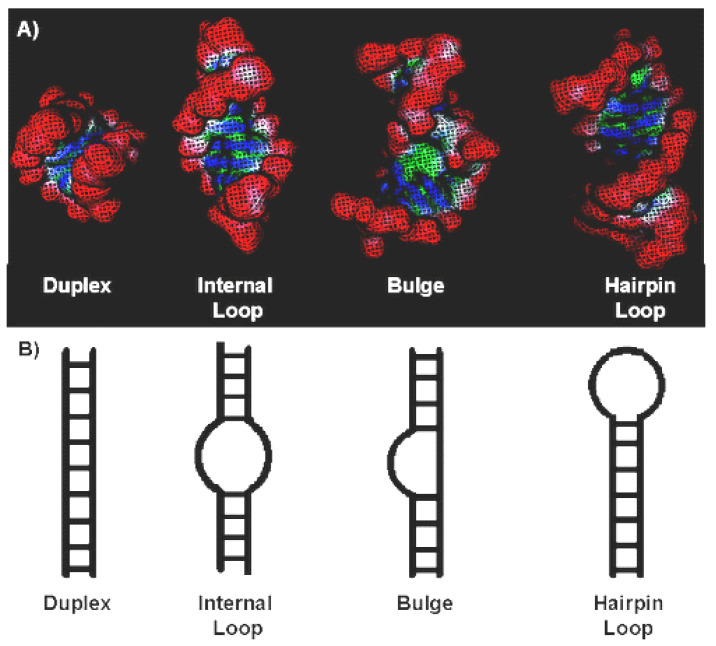
Four general classes of RNA secondary structure. (**A**) Surface representations of individual secondary structures. (**B**) Schematic representation of RNA duplex, internal loop, bulge, and hairpin loop (also called stem loop) regions of RNA [82].

In terms of targeting, RNA has many attractive properties similar to those of DNA and/or proteins. Both forms of nucleic acids take a regular helix with large and small grooves. However, the usual helix of the A-form of RNA is often disturbed by regions of inappropriate or unpaired bases; they lead to a change in the secondary structure of RNA (Figure 13).

Compared with the small and large grooves of DNA, these structural motifs in RNA are much smaller, since the pitch of the helix and the inclination of the bases are smaller. According to [83,84], the width of the main groove of the A-shape helix is only 4 Å and this circumstance excludes the binding of small organic compounds, including P&A. Thus, the targets available for binding to the P&A are the duplex RNA regions presented above (Figure 13). During the interaction of P&A with duplex RNA, as in the case of DNA, the formation of 2 types of complex is possible—external with self-stacking P&A and intercalation.

The first publication on the interaction of cationic TMPyP4 porphyrin with RNA duplexes dates back to 1997 [85]. A similar approach based on the interaction of cationic porphyrins with mRNA G-quadruplexes is used to evaluate the use of porphyrins in photodynamic therapy [86]. The introduction of copper ion into the TMPyP4 reaction center leads to the binding of CuTMPyP4 to rA·rU base pairs in duplex RNAs. The introduction of metal contributes to the formation of stronger complexes with RNA duplexes compared with similar complexes of metal-free porphyrin [87]. There are also known data on the interaction of RNA G-quadruplexes with phthalocyanines [88].

A series of works is devoted to MD modelling of the binding of TMPyP4 to RNA and oligonucleotides [85,89], the result of which was the conclusion about the binding of TMPyP4 in large and small grooves and an unusual external binding in which porphyrin is positioned so that its plane is oriented parallel to the main chain of the biopolymer. Similar schemes of complexes contradict the existing views [82,90], whereas another work [91] of the same authors reports on the intercalation binding of TMPyP4 to a synthetic polynucleotide poly(adenyl), poly(uridyl) acid (Poly(A)-Poly(U)).

**Figure 14:**
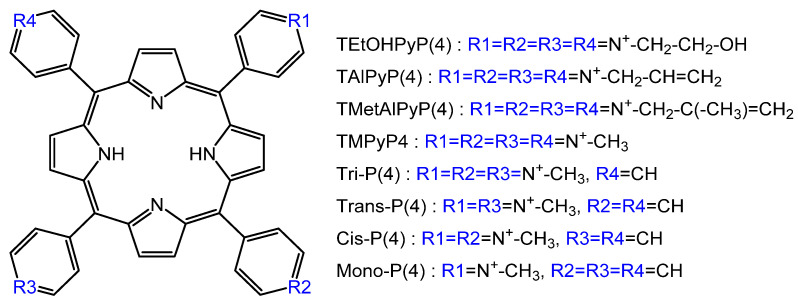
Structures of the P&A [93].

The effect of the modification of the cationic porphyrin substituent (Figure 14) on the binding to poly(rA) poly(rU) and poly(rI) poly(rC) homopolymer RNA duplexes was studied in [92].

TEtOHPyP4 and TAlPyP4 intercalate into duplexes poly(rA) poly(rU) and poly(rI) poly(rC), whereas TMetAlPyP4 binds to these RNA duplexes, forming an external complex with self-stacking [92]. It is noteworthy that from the spectral data obtained in the temperature range of 18 and 45 °C, the authors determined the thermodynamic parameters of binding [92], while not taking into account that the van’t Hoff approach is not acceptable for thermosensitive biopolymers and their complexes with a large fraction of the electrostatic contribution.

As a rule, P&A compounds form not very strong complexes with RNA due to structural factors, therefore, chemotherapeutic agents for the inactivation of RNA viruses have not yet been proposed. On the other hand, there remains the possibility of photoinduced inactivation of RNA viruses at the stage of transcription and replication of the viral genome. As shown above, in the case of DNA, all four bases are oxidized, but guanine is the most susceptible; it produces 8-oxo-7,8-dihydro-2′-deoxyguanosine under the influence of singlet oxygen. It is this compound that is often used as a marker of oxidative damage in DNA [94]. In the case of RNA, there is no such marker. Confirmation of RNA photooxidation is carried out using microbiological and analytical methods. However, there are certain difficulties, as many RNA viruses are uncultivated or difficult to cultivate (e.g., hepatitis A, human norovirus, etc.). Analytical methods (PCR) in the case of (−)RNA imply pretreatment with reverse transcriptase, which may be accompanied by a large number of errors [95]. These factors limit the study of photoinactivation of viral RNA. There is relatively scarce evidence that inactivation of RNA viruses with photo-irradiation of the P&A is possible, and it can lead to mutations in the genome. Oxidized RNAs can lead to the synthesis of defective proteins that are not able to form a capsid or provide a binding to the host cell receptor [96].

It is the violation of the biochemical functions of RNA during photoirradiation of the external stacking complexes of *meso*-tetrakis(4-*N*-methylpyridyl)porphin, *meso*-tetrakis(para-*N*-trimethylanilinium)porphin, and *meso*-tetrakis(2-*N*-methylpyridyl) porphin and RNA and complexes of porphyrins with elements of the secondary structure of RNA [97] that causes inactivation of the HIV-1 virus. From a chemical point of view, the photoinduced reaction of ^1^O_2_ with viral RNA leads to the modification of nucleosides in the region of localization of porphyrins (inner and stem loops and hairpins, Figure 13). Chemical modification of these areas of the secondary structure of RNA conditions a change in the tertiary structure, namely, a change in coaxially laid RNA helices [97].

Thus, the formation of complexes between the P&A and DNA/RNA viruses can result in chemical and/or photochemical inactivation. Currently, the possibility of inactivation of viruses in vitro and in vivo is mainly determined by the mechanism of chemical inactivation. In this case, there is a potential danger, since the chemical modification of the genome may not inactivate the virus, but lead to a mutation [98]. The main difficulty in using P&A to inactivate viruses with a molecular target (the genetic material of viruses) lies in the low selectivity of P&A and the active binding of P&A to DNA by host cells.

Perhaps, the solution to this problem is not far off. A chemical approach to DNA recognition [99] based on the use of polyamides has already been found. Analogues of the *N*-methylpyrrole rings of these polyamides provide a set of five-membered heterocycles that can be combined (in the form of asymmetric pairs of rings) in a modular manner to recognize predetermined DNA sequences with affinity and specificity comparable to DNA-binding proteins. The presence of such a peripheral substituent in the P&A can solve the problem of precise targeting of viral DNA.

**Figure 15:**
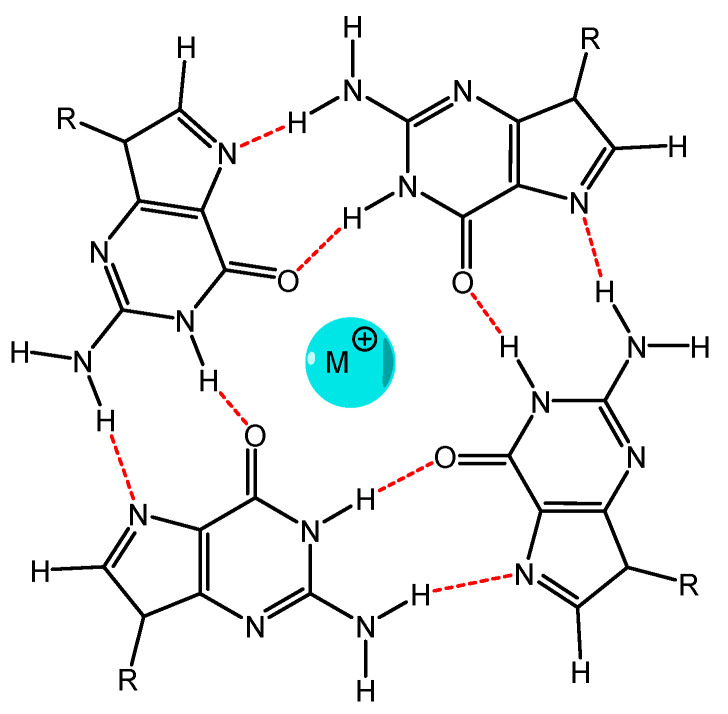
The G-quadruplex structure.

Another possible solution to the problem of selectivity for targeting the P&A to the viral genome is to accurately target the sections of the genetic material of viruses, such as, e.g., G-quadruplexes. Guanine-rich RNA or DNA sequences are capable of folding and accepting four strands, called G-quadruplexes or “G4” (Figure 10E). Unique features of the G4 topology, very different from DNA duplex or single-stranded RNA, make it a potential therapeutic target. The orientation of the strands determines a parallel, antiparallel, or mixed topology of G4, and it is directly related to the conformational state, the anti- or syn-glycosidic bond between the base G and sugar (Figure 15) [100].

G4 structures are widely represented in the human genome, especially in telomeres and oncogenic promoters. In recent years, the presence of G4s in viruses has attracted increasing interest. G-quadruplexes were found in several viruses, including those associated with recent epidemics, such as Zika and Ebola viruses, SARS, and COVID-19 [101]. Viral G4s are usually located in regulatory regions of the genome and are involved in the control of key viral processes, such as virulence, virus replication, recombination, and structural changes to evade the immune response [102]. Comprehensive information on the number of G-quadruplexes and their functions in viruses is presented in reviews [102,103,104]. Inactivation of viruses is possible due to the formation of a strong complex between the P&A and G-quadruplexes of viruses. When designing porphyrins and their analogues, it is important to achieve high stability of their complexes with G-quadruplexes, and it is possible with strict chemical and geometric compliance. Cationic porphyrins TMPyP4 and (5,10,15,20-tetrakis-(*N*-methyl-2-pyridyl)porphin) (TMPyP2) were proposed as a complexing compound for G4 of a number of viruses [105]. TMPyP4 compared with the TMPyP2 isomer showed the best results [106] in inactivation of HIV-1 virus. After incubation with TMPyP4, the expression of Nef protein, the main necessary protein for virus replication, was reduced. The authors suppose that it occurred due to the formation of a stable complex between TMPyP4 and G-quadruplex structures in the nef HIV-1 coding region [105]. It is interesting to note that the stability of the resulting complexes, on which the antiviral activity of porphyrin depends, was evaluated according to the melting points of free and complexed G-quadruplex. Later, tetracationic porphyrins exhibiting antiviral activity against different strains of HIV were patented [107]. It is noteworthy that exploratory studies are carried out in the direction of modifying cationic peripheral substituents [108], but it is overlooked that the complex of G-quadruplexes with P&A is stabilized not only due to anion–cationic interactions but also due to the extended π–π-interaction between the aromatic system of porphyrin and guanine bases.

The cationic porphyrins TMPyP2, TMPyP3, and TMPyP4 were considered as potential binding agents for Epstein–Barr virus G-quadruplex RNA. It turned out that TMPyP3 and TMPyP4 form a stable complex with G regions of the virus genome, and it does not allow to encode the EBNA1 protein, which is crucial for replication and maintenance of the genome during latency in proliferating cells [109].

In numerous studies, it was shown that cationic porphyrins containing peripheral charged substituents in the para position, as a rule, demonstrate greater efficiency compared to both the ortho- and metaisomers. In this sense, the approach of the authors [110] comparing the antiviral activity of the TMPyP2 mesoisomer and the chemotherapeutic agent of BRACO-19 (trisubstituted acridine, characterized by excellent G-quadruplex binding properties) against herpes simplex virus (HIV-1) is not clear as well as their conclusion about the absence of the P&A study prospects in general.

TMPyP4 was also used to study the role of G4s in EBOLA virus replication [111]. For this, the genetic materials of the original virus and the mutant that does not contain G4 quadruplexes in the L gene were studied. It was established that TMPyP4 exhibited high stabilization of only the initial RNA. More important is that after treatment with elevated concentrations of TMPyP4, gene transcription gradually decreased.

If we compare the affinity of TMPyP4 with respect to G4-structures and duplex DNA, then it will be lower [112], respectively, and the antiviral activity of TMPyP4 can have several mechanisms of action and they limit its biological and clinical use.

It is quite remarkable that in the vast majority of works on the inactivation of viruses, cationic porphyrins are considered as a binding agent for G-quadruplex structures. Although, as shown in [113], anionic P&As are also capable of binding to G structures, moreover, more selectively.

And if the issue of the selectivity of virus inactivation in living organisms remains open, then one can definitely say that recently successful attempts have been made to use P&A to reduce the transmission of pathogenic microorganisms during blood transfusion. Viral infections, the potential risk of their transmission, narrowly targeted viral tests, and the lack of adequate and systematic screening for the virus and bacteria in the blood and red blood cells make the problem of increasing blood products safety very urgent. Impressive results were demonstrated by the authors of [93] by the example of inactivation of the vesicular stomatitis virus in red blood cells for blood transfusion. To evaluate virucidal and erythrocyte-damaging activity, a number of cationic porphyrins were considered (Figure 14).

The most effective photosensitizer from the tested series, which at the same time maintained the integrity of red blood cells, was Tri-P (4). It caused the least hemolysis under conditions that led to a 5-fold decrease in the extracellular virus of vesicular stomatitis. All positively charged tested porphyrins were able to inactivate the extracellular virus of vesicular stomatitis, but caused a violation of the integrity of red blood cells during storage. Vesicular stomatitis virus is a single-stranded lipid RNA virus. Unfortunately, the authors did not advance in the analysis of the “structure (porphyrin)-property (virucidal ability),” but they only suggested that the cause is the interaction of porphyrins with RNA. Judging by the data presented [93], the virucidal activity decreases in the series: Tri-P(4) < TEtOHPyP(4) < *Trans*-P(4) < *Cis*-P(4) < TMPyP4 < Mono-P(4). Moreover, the last three porphyrins caused the maximum destruction of red blood cells. Obviously, in addition to the photochemical properties of the photosensitizer, penetration (through the lipid membrane) and binding to RNA are significant.

In conclusion, we would like to note that the chemo- and photoinactivation of P&A in viruses is very promising and can become an alternative and highly effective way to treat viral infections. To increase the selectivity of the action of P&A on viral targets, a targeted modification of the structure of P&A is necessary. The complexity and versatility of the objects of research emphasize the need to combine the efforts of chemists, microbiologists, and virologists.
